# Analysis of common methodological flaws in the highest cited e-cigarette epidemiology research

**DOI:** 10.1007/s11739-022-02967-1

**Published:** 2022-03-24

**Authors:** Cother Hajat, Emma Stein, Arielle Selya, Riccardo Polosa, The CoEHAR study group, Salvatore Alaimo, Carmelina Daniela Anfuso, Ignazio Barbagallo, Francesco Basile, Sebastiano Battiato, Brahim Benhamou, Gaetano Bertino, Alberto Bianchi, Antonio G Biondi, Maria Luisa Brandi, Emma Cacciola, Rossella R Cacciola, Bruno Santi Cacopardo, Aldo E Calogero, Maria Teresa Cambria, Davide Campagna, Filippo Caraci, Agatino Cariola, Massimo Caruso, Pasquale Caponnetto, Adriana Ciancio, Fabio Cibella, Maurizio di Mauro, Jennifer di Piazza, Adriana di Stefano, Filippo Drago, Salvatore Failla, Rosario Faraci, Salvatore Ferlito, Margherita Ferrante, Alfredo Ferro, Giancarlo A Ferro, Francesco Frasca, Lucia Frittitta, Pio M Furneri, Antonio Gagliano, Giovanni Gallo, Fabio Galvano, Giuseppe Grasso, Francesca Guarino, Antonino Gulino, Emmanuele A Jannini, Sandro La Vignera, Giuseppe Lazzarino, Caterina Ledda, Rosalia Maria Leonardi, Giovanni Li Volti, Antonio Longo, Gabriella Lupo, Mario Malerba, Luigi Marletta, Guido Nicolosi, Francesco Nocera, Gea Oliveri Conti, Giuseppe Palazzo, Rosalba Parenti, Eugenio Pedullà, Alfredo Pulvirenti, Francesco Purrello, Francesco Rapisarda, Venerando Rapisarda, Renata Rizzo, Simone Ronsisvalle, Giuseppe Ronsisvalle, Martino Ruggieri, Maria C Santagati, Cristina Satriano, Laura Sciacca, Maria Salvina Signorelli, Marco Tatullo, Daniele Tibullo, Venera Tomaselli, Vladislav Volarevic, Luca Zanoli, Agata Zappalà

**Affiliations:** 1 Public Health Institute, UAE University, Al Ain, United Arab Emirates; 2 New Haven, USA; 3 Pinney Associates, Inc, Pittsburgh, PA USA; 4 Behavioral Sciences Group, Sanford Research, Sioux Falls, SD USA; 5 Department of Pediatrics, University of South Dakota Sanford School of Medicine, Sioux Falls, SD USA; 6 Center of Excellence for the Acceleration of HArm Reduction (CoEHAR), University of Catania, Catania, Italy; 7 Department of Clinical and Experimental Medicine, University of Catania, Catania, Italy; 8 Institute of Internal Medicine, AOU “Policlinico-V. Emanuele”, Via S. Sofia, 78, Catania, Italy

**Keywords:** e-cigarettes, Vapes, Vape pens, Electronic nicotine delivery systems (ENDS), Epidemiology, Critical Analysis

## Abstract

The prevalence of vaping, also known as using e-cigarettes, vapes and vape pens, has prompted a demand for reliable, evidence-based research. However, published literature on the topic of vaping often raises concerns, characterized by serious flaws and a failure to adhere to accepted scientific methodologies. In this narrative review, we analyze popular vaping studies published in medical journals that purport to evaluate the association of vaping and smoking cessation, smoking initiation or health outcomes. We analyzed 24 included studies to identify the questions they claimed to address, stated methods, manner of implementation, discussions, and stated conclusions. After critical appraisal, we noted a multiplicity of flaws in these studies, and identified patterns as to the nature of such flaws. Many studies lacked a clear hypothesis statement: to the extent that a hypothesis could be inferred, the methods were not tailored to address the question of interest. Moreover, main outcome measures were poorly identified, and data analysis was further complicated by failure to control for confounding factors. The body of literature on “gateway” theory for the initiation of smoking was particularly unreliable. Overall, the results and discussion contained numerous unreliable assertions due to poor methods, including data collection that lacked relevance, and assertions that were unfounded. Many researchers claimed to find a causal association while not supporting such findings with meaningful data: the discussions and conclusions of such studies were, therefore, misleading. Herein, we identify the common flaws in the study design, methodology, and implementation found in published vaping studies. We present our summary recommendations for future vaping research. Our aim is to prompt future researchers to adhere to scientific methods to produce more reliable findings and conclusions in the field of vaping research.

## Introduction

Health policy on vaping, also known as using e-cigarettes, vapes, vape pens, or electronic nicotine delivery systems (ENDS) [1], should be guided by scientific research. However, the majority of published studies on the topic of vaping are replete with flawed methodologies, misleading discussions, and unreliable conclusions [2]. Misleading literature can misinform well-intended health care practitioners, researchers and policy makers, as well as patients and caregivers. It is, therefore, essential that published literature on the topic of vaping be reviewed to establish whether they are fit for this purpose.

As many journal articles on the topic of vaping or tobacco smoking provide conflicting and unsubstantiated research findings, we undertook a critical appraisal of such research articles. Herein, we delineate our findings, including common flaws in study design, participant recruitment, data analysis, and other methods that undermine the reliability of vaping studies. The purpose of this paper is threefold: (i) to help guide researchers who endeavor to improve the quality of study design and methods; (ii) to prepare readers to critically evaluate the reliability of vaping research and literature; and (iii) to address myths and misconceptions perpetuated by flawed vaping literature.

## Methods

We used the Google Scholar search engine (30 November 2020) to obtain the most "popular" journal articles on vaping research. We used the Google algorithm definition of "popular", i.e., the articles most read and most cited in other literature and policy discussions. We searched behavioral human subjects research on causal claims related to vaping. Specifically, we ran the search string: “e-cigarette OR ‘electronic cigarette’ OR vaping OR ‘electronic nicotine delivery system.’" One researcher stepped through the articles in order of search results ranking and identified the ten most frequently cited articles on each of the following topics: (i) the effects of vaping on smoking cessation/reduction; (ii) the effects of vaping on smoking initiation; and (iii) the health outcomes associated with vaping. A second researcher reviewed the ten identified studies for inclusion. Disagreements between the two reviewers were resolved by consensus or by the decision of a third reviewer, and additional studies were stepped through until ten agreed upon studies were identified.

We acknowledge that alternative methods exist to define "popular" and to identify vaping literature, such as a PubMed search. However, we used the Google Scholar algorithm for the purpose of this paper because it better reflects the search methods used by policy makers, advocacy groups, health care providers, and patient populations.

An initial search returned the titles of articles, which, upon manual review, were determined to not truly meet search criteria. Such articles were replaced by continuing through the search. Excluded papers were those that did not meet our intended search criteria, e.g., those that addressed descriptive epidemiology, chemistry and toxicology, acute responses to exposure, and analytic papers that were not empirical.

We conducted a review and critical appraisal of the 24 most popular journal articles on causal claims related to vaping and discuss our findings below. An analysis of each paper includes a discussion of common study design and methodology flaws. In particular, we critically analyzed papers for significant limitations: improper methods; significant flaws in applying potentially useful methods; suboptimal participant recruitment and retention. Specifically, papers meeting inclusion criteria were critically analyzed for the following strengths and limitations:

1. Did the study clearly describe the method of investigating causal pathways? Scientific standards require researchers to specify a causal hypothesis, and describe a study design and data collection methods to investigate that hypothesis. If researchers merely discuss a causal association and present statistical data without establishing causation, we highlight such deficiencies.
2. Were the study design and research methods sufficiently robust to control for confounding factors?
3. Do the results support the stated conclusions, without overstatement?
4. Do the researchers present language or data that is misleading, or fail to acknowledge significant limitations?

While many included papers contained idiosyncratic problems, we did not address such flaws as they fell beyond the scope of our analysis. Instead, we highlight the themes of common flaws that warrant focused attention that will guide future researchers.

One researcher then grouped the studies according to whether they addressed the effects of vaping on smoking cessation and reduction; the effects of vaping on smoking initiation; or of vaping on health outcomes (Table 1) A second researcher critically appraised studies and reported on each study, presenting strengths and limitations (Appendices A, B and C), which were discussed with the other researchers until a consensus was reached on each study.

**Table 1:** Main characteristics of included studies

Sources	Citations	Study design	Outcomes of interest assessed		Country
			Potential effect of vaping on	Epidemiology of smoking	
Alzahrani et al. [4]	172	Cross-sectional	Myocardial infarction	Yes	US
Barrington-Trimis et al. [5]	61	Three pooled cohorts	Smoking initiation	–	US
Beard et al. [6]	34	Repeated cross-sectional	Smoking initiation	–	UK
Bhatta et al. [7]	62	Cross-sectional	Myocardial infarction	Yes	US
Biener et al. [8]	358	Longitudinal	Smoking cessation/reduction	–	US
Bold et al. [9]	125	Longitudinal	Smoking initiation	–	US
Brown et al. [10]	215	Cross-sectional	Smoking cessation/reduction	–	UK
Etter et al. [11]	286	Longitudinal	Smoking cessation/reduction	–	US, UK, Switzerland
Giovenco et al. [12]	114	Cross-sectional	Smoking cessation/reduction	–	US
Gmel et al. [13]	51	Longitudinal	Smoking cessation/reduction	-	Switzerland
Goldenson et al. [14]	94	Prospective cohort	Smoking initiation	–	US
Gomajee et al. [15]	30	Cohort	Smoking cessation/reduction	–	France
Grana et al. [16]	309	Longitudinal	Smoking cessation/reduction	–	US
Hitchman et al. [17]	266	Cross-sectional	Smoking cessation/reduction	–	UK
Leventhal et al. [18]	115	Longitudinal	Smoking initiation	–	US
Levy et al. [19]	97	Cross-sectional	Smoking initiation	–	US
Martinez et al. [20]	22	Cross-sectional	Smoking cessation/reduction	–	US
McConnel et al. [21]	203	Cross-sectional	Respiratory symptoms	Yes	US
Miech et al. [22]	160	Longitudinal	Smoking initiation	–	US
Primack et al. [23]	477	Longitudinal cohort	Smoking initiation	–	US
Spindle et al. [24]	145	Longitudinal	Smoking initiation	–	US
Unger et al. [25]	130	Longitudinal	Smoking initiation	–	US
Warner et al. [26]	87	Cross-sectional	Smoking cessation/reduction	–	US
Wills et al. [27]	85	Cross-sectional	Respiratory disorder	Yes	US

Moreover, to avoid any misjudgment of the selected studies, we contacted each corresponding authors and shared the critical appraisal of their papers asking them to identify misinterpretations from our part and to discuss any additional methodological limitations/strengths of their work. In all cases, we received constructive advice that improved the quality of our final analyses and appraisal.

**Fig. 1:**
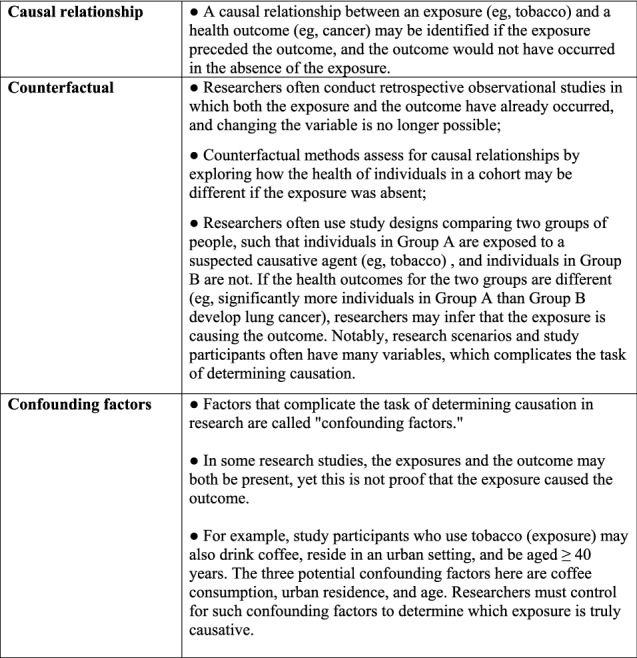
Terminology related to assessing for causal associations between vaping exposures, behavior, and health outcomes

We also analyzed the studies collectively as a body of literature, highlighting common missteps in study design, methodology, and implementation (Table 2). As many included papers demonstrated common research flaws involving confounding factors, causative associations, and the counterfactual analysis [2, 3], these terms are set forth in Fig. 1.

**Table 2:** Included studies: study design, methodology, and implementation flaws

Consideration	Methodological issue	Observations from the review
Hypotheses, counterfactuals, and causation		
Hypothesis statement	State causal hypothesis at outset of research document. Relate study design and methods to hypothesis. Distinguish associations that are causal from those that are not	Many included papers lacked clear hypothesis statements. Many included papers contained vague conclusory statements unconnected to a focused hypothesis or data analysis
Reporting results	Relate results to hypothesis clearly and specifically	Included papers make causal claims that are unreliable because they fail to specify which health outcome/exposures are the subject of the conclusion. They also offer conclusions and summary statements unsupported by data
Counterfactual analysis	Researchers state a hypothesis but fail to state a counterfactual claim that could guide data analysis and clarify interpretations	In the included papers the authors often fail to state their hypothesis clearly and have not discussed counterfactual claims
Causal pathway identification	Identifying and specifying the suspected causal pathway is needed to explain why "reverse causation" is not plausible. It is also needed to determine which variables are potential confounders, and assess for their impact on causation	The included papers lack clear statements of the suspected casual pathway. They also discuss covariates without specifying their relevance to the causal pathway or potential confounding
Asserting causal inference	A causal inference may be drawn if a health outcome would not have occurred in the absence of the candidate cause. Multiple candidate causes may contribute to a single health outcome. Each candidate cause should be considered in the data analysis	The included papers discuss associations between exposures and outcomes, but do not analyzing data to establish causation
Human subjects research principals		
Participant perspectives	Input from study participants should be solicited to inform researchers and enhance data gathering. Notably, individuals who vape have published voluminous relevant content. Researchers often actively solicit input from the population of interest before formulating their study design and recruiting participants	Included papers demonstrate a lack insights and context that could have been obtained from participants. They use language that demonstrates disregard for participants
Researcher insights	Researchers should have a basic understanding of the behavior studied (e.g., usage patterns, methods, motivations, product choices)	Several of the included papers in the cessation and uptake categories demonstrate inadequate knowledge of topics (e.g., distinctions between vaping and smoking behaviors; motives underlying vaping system choice)
Cultural awareness and sensitivity	Researchers are expected to demonstrate respect for study participants, demonstrate cultural competence, and use appropriate language	In the included papers, authors demonstrate low levels of cultural competence, disregard for study participants, and use demeaning language
Relevant metrics: focus population, time, and outcome measures		
Definition: exposure	Definitions of exposure for vaping studies (e.g., daily, one puff in last 30 days, every tried) should be tailored to the hypothesis and consistent with metrics in related research	In the included studies, the metrics and main outcome measures appear to have been selected arbitrarily. Metrics and data are not related to the hypothesis or appropriately addressed in a discussion about causation. There is little consistency across studies
Generalizability	Study conclusions are limited if they are not generalizable to diverse participant populations as to time, place, and other characteristics Studies of the impact of vaping on smoking are difficult because they assess for behavioral change in a population with a high proportion of people already undertaking that behavioral change (i.e., smoking to vaping). They also must control for factors influencing behavior such as societal fads, youthful caprice, and the allure of rapidly changing technology	The included papers lack context for the behavioral activities addressed. The metrics and outcome measures are unlikely to produce generalizable results
Representative sample: participants	Researchers should explicitly discuss results and conclusions in the context of the participant population. The extent to which results cannot be extended beyond such populations should be specified	Most included studies failed to acknowledge the limited generalizability of their findings beyond their participant population
Switching: tobacco-related behaviors	Time trends in switching behavior are likely to occur, with the population of potential switchers being depleted. Thus switching rates will not be constant but rather will be affected by some expected attrition	In the included studies, the researchers do not account for this trend
Effect of vaping on smoking cessation: flawed research methods		
Stock-flow problem	Many studies attempt to measure the proportion of people who quit smoking, i.e., the "flow" of people. However, many people who have already quit smoking successfully due to vaping are excluded from studies. Thus, the impact of vaping on smoking cessation is not effectively measured in many studies due to inclusion/exclusion criteria For many candidate exposure measures, the "stock" of people who vape comprise a disproportionately high number of people who are not likely to switch, and exclude those who previously quit smoking by switching. A study cohort with such a skewed proportion of participants creates bias in the study	In all included studies, the "stock-flow" problem is a serious limitation. While some of the researchers included steps to mitigate the impact of the stock-flow problem, it remained significant and unacknowledged
Impact of smoking cessation on vaping behaviors	Smoking cessation causes particular vaping behaviors (e.g., increase in vaping frequency to daily use; purchasing vaping paraphernalia), irrespective of whether or not those behaviors impact smoking cessation	The authors who looked at these variables did not acknowledge this issue
Other potential biases: longitudinal studies	A longitudinal cohort study design is potentially more informative than other study designs; however, a case–control study design may be more informative to research the topics of vaping and smoking cessation. The overall study design, methods, and data collection, implementation, and interpretation are essential to determining the value of the study	The included studies reveal multiple methodology flaws such as the stock-flow problem, cross-sectional surveys of subjects, with no meaningful retrospective questions
Retrospective and motivational questions	Retrospective studies comprising participant questionnaires are informative only to the extent that meaningful questions elicit accurate information on relevant metrics	The retrospective studies included do contain some meaningful questions; however, the overall study designs are not sufficiently comprehensive to produce reliable responses or data
Effect of vaping on smoking initiation: flawed research methods		
Confounding: individual propensities	Confounding may occur because many participants engage in multiple behaviors (e.g., use of illicit drugs, tobacco use, vaping of nicotine and/or cannabis) which may or may not be reported. Such variables warrant assessment to determine association(s) with main outcome measures, and whether any associations are causative	The included studies discussing "gateway" behaviors do not adequately address confounding variables, and therefore, do not reliably discuss causation
Intractable confounding	Research reveals heterogeneity among those who vape and/or smoke as to an essential metric: some individuals like consuming nicotine and some do not. Studies must distinguish participants according to these traits	In the included studies, researchers fail to design studies that recognize the heterogeneity of participants according to the key trait of nicotine preference
Gateway theory	The "Gateway" theory posits that a person who vapes is more likely to begin using other substances (e.g., illicit drugs, cannabis, smoking cigarettes) than someone who never vaped The "Gateway" theory is often stated as a forgone conclusion—but must be supported with data and reliable research Unsupported assertions of a "Gateway" theory warrant skepticism, as the other behaviors of concern have significant barriers to adoption (e.g., more harmful, less flavorful, less convenient, and more expensive)	The included studies embracing the "Gateway" theory do so without scientific support
Evidence-based methodology		
Methodology flaws	For research to be reliable, sound methods are required, including recruiting and retaining an adequate number of participants, accurate measurements, and reasonable follow-up	The included studies reveal many preventable methodology flaws, including small sample size, poor participant follow-up and retention, and unreliable measurements
Small effect size	The main outcome measure of the study should be measurable and salient. If potential confounders obscure researchers' ability to assess the main outcome, the study will be uninformative	Many of the included studies addressing health effects attempt to discuss an outcome measure that is too small to have been meaningfully assessed
Real-world science	If research findings conflict with real-world, common sense observations, the researchers should explain this apparent inconsistency	In many included papers, authors assert that vaping reduces the likelihood of smoking cessations and/or promotes smoking initiation. Such assertions are not supported by reliable research and are contrary to real-world observations

## Results

The 24 journal articles identified by our search methods are listed in Table 1 [4–27]. Many papers purporting to be scientific literature contained only subjective information. Exclusions included approximately 28% of search results that addressed cannabinoid vaping (EVALI) or other cannabis use, and approximately 33% of search results that were case studies.

The majority of papers devote their focus to either smoking cessation or initiation, not both; such articles are assigned to one category accordingly, even if the article contains secondary discussion of the other topic (Table 1). One paper included a substantial analysis of both smoking cessation and smoking initiation and is addressed in both sections (Table 1). The remaining papers address the health outcomes associated with vaping (Table 1).

The 24 included papers for all three categories were riddled with flaws as summarized below and discussed in more detail in Table 2 and Appendices A, B, C.

### Effects of vaping on smoking cessation and reduction

The ten articles on the effect of vaping on smoking cessation or reduction are described in more detail with regards to strengths and limitations in Appendix A. An individual who smokes cigarettes may engage in vaping as a strategy to aid smoking cessation or reduction. Several research studies purport to assess the effect of vaping on smoking cessation and reduction success. A critical appraisal of these studies revealed numerous flaws. Researchers often evaluate the probability of success for a given quit method, yet mistakenly assume that the number of quit attempts is fixed. In fact, education as to a novel quit strategy may prompt additional quit attempts. Thus, the quit method (e.g., vaping) warrants credit for prompting an additional quit attempt. Research study designs should include a multivariate analysis, and control for confounding, assessing factors such as vaping status, smoking status, cessation and reduction goals, number and method of quit attempts.

Several researchers failed to clearly state the causal pathway they were investigating. For example, if an individual has a successful smoking quit attempt, and would not have successfully quit in the absence of vaping, then vaping caused that cessation. However, other potential causal pathways exist that the researchers did not explore. Consider the individual who would not otherwise have made a quit attempt, yet does so (and succeeds) because the option of vaping motivates the quit attempt. This second pathway includes both intentional quit attempts, and unintentional quitting (dubbed, the “accidental quitter” phenomenon), whereby someone who smokes tries vaping without the intention of switching, but finds it so appealing that they switch.

The researchers also selected flawed inclusion/exclusion criteria. For example, a given population may consist of a many former smokers who have made successful quit attempts by switching to vaping. To conduct a study in such a population, but include only active smokers, researchers will be evaluating only those already less likely to quit by switching (as suggested by the fact that others did, and they did not). Moreover, to exclude former smokers who successfully quit by vaping creates a biased participant population. In our literature review of vaping effects on smoking cessation, many researchers did not account for these trends. The numerous anecdotal reports of those who found vaping to be an effective aid to smoking cessation may inspire future researchers to formulate robust study designs and participant recruitment methodology.

Epidemiological studies assessing population trends may note the incidence and prevalence of vaping and smoking. However, such studies generally lack the specificity needed to establish a causal association between vaping and smoking cessation. Moreover, incidence and prevalence of vaping-related behaviors tend to fluctuate due to a confluence of variables, such as changing technology, marketing, and media coverage. Research on general population trends should include relevant data points in their analysis and not overstate their conclusions.

### Effect of vaping on smoking initiation

The effect of vaping on smoking initiation was addressed in 11 of the included papers, with detailed discussion of strengths and limitations in Appendix B.

One risk from vaping investigated by researchers is the possibility that those who initiate vaping are more likely to subsequently initiating smoking. Often dubbed a “gateway effect,” this potential causal association is often asserted as if proven by data, when it is not.

The flaws in studies addressing the “gateway effect” have been discussed at length [28]. The studies we analyzed lacked sound research methods, and as such, could not reliably establish causation or identify a gateway effect. Moreover, health behaviors related vaping and smoking were described with insufficient detail as to the duration, amount, and frequency of the vaping/smoking. This renders participant classification uninformative, and the resulting data unreliable. For example, the phrases “tried vaping” and “was *a vaper*” may describe two very different levels of vaping exposure, yet these participants may be classified together in a research study. Moreover, researchers should be sufficiently culturally competent to explore causal pathways. For example, “tried vaping, discovered an appreciation for nicotine, and as a result took the opportunity to start smoking when it was presented” is a plausible causal pathway, whereas “became a dedicated vaper and then switched to smoking” might represent someone who would have become a smoker anyway.

Further, the propensity to initiate tobacco use in the absence of vaping is also poorly established, and as such, constitutes a questionable metric. This is particularly troublesome when the claims of a “gateway effect” may be exploited, without support, to create concern regarding other risk taking behaviors, such as illicit drug use. Discussions in health literature should be grounded in data and not be unduly alarmist.

Further, it is important to categorize participants according to nicotine preference: about half the population likes being under the influence of nicotine and half does not. This variation alone guarantees a substantially higher smoking uptake among vapers (and vice versa). This is partially a result of physiology and psychological characteristics, and partially a matter of attitude. Most people who do not use any nicotine product are actively averse to doing so. Thus, people who never vape, smoke, or use any other any tobacco product will inevitably initiate one such product less often than users of tobacco products.

The studies analyzed did not control for confounding or use methods designed to account for heterogeneity among participants. As such, the limitations were significant, and the researchers could not credibly make causal claims. Finally, many studies contained data findings suggesting that vaping behavior may replace would-be smoking. Populations studies reveal trends of increased rates of vaping associated with decreased rates of smoking. However, the role of vaping in preventing smoking initiation has not been fully investigated, and is a meaningful topic for future research.

### Epidemiology of smoking, vaping, and health outcomes

The four papers that addressed health outcomes from vaping (Appendix C) also had numerous flaws. For example, researchers attempted to assess the non-acute effects of vaping in a population of former smokers without acknowledging an inherent limitation: the characteristic clinical traits of this population include the consequences of prior smoking, which mask the non-acute effects of vaping. In such a population, it is difficult to determine whether morbidity and mortality outcomes are attributable to vaping or prior smoking. It is a major design flaw to fail to account for current, former and dual use of cigarettes, thereby ignoring that the majority of vapers do so to quit or cut down on cigarette smoking [29–31]. Despite up to 70% of e-cigarette users reporting dual use, 32 studies did not routinely account for dual use when investigating risk from vaping, thereby attributing health outcomes to vaping when they may have actually resulted from smoking cigarettes. It is also difficult to design a study and identify potential participants to control for likely confounding factors.

There is ample evidence to suggest that the individual chemical exposures from vaping cause either a fraction of the risk posed by smoking. The plausible range here is an order of magnitude smaller than the variation in the residual health effects from former smoking, which vary based not merely on the existence of former smoking (typically the sole metric), but other factors, e.g., the duration and quantity of former smoking (occasionally measured), time since quitting (occasionally measured), intensity of use and puffing behavior (rarely measured). Classifications of smoking status also lacked granularity. For example, many studies merely classify smoking status generally (e.g., current, former or never) without accounting for duration of smoking, time since quitting, or frequency and quantity of tobacco use.

These flaws were found consistently in each of the four articles analyzed, rendering their conclusions misleading. Long-term prospective studies of appropriately categorized participants would be useful to compare the health outcomes associated with vaping to those of smoking. It is also essential to control for confounding. In conclusion, the use of unrefined definitions and classifications in this population is a serious flaw in study design and methodology.

In Table 2, we provide a summary of our critical appraisal revealing common, preventable flaws, the identification of which may guide future researchers to improve on their ability to design or appraise not only tobacco harm reduction research domain, but any form of epidemiologic research for that matter.

## Discussion

A critical review of the included literature revealed numerous flaws, and limitations notably outweighed strengths.

For articles on smoking cessation and reduction, most notably the researchers failed to acknowledge that vaping as a quit strategy may increase the number of quit attempts, thereby increasing the likelihood of success. Further, many studies lacked a robust design with a multivariate analysis that controls for confounding. Moreover, the researchers often failed to articulate a hypothesis or identify a suspected causal pathway. Finally, the researchers used flawed inclusion/exclusion criteria for study participants, such that former smokers who already quit using vaping as a quit strategy are excluded, effectively reducing the number of people who found this method successful.

Many researchers investigating smoking initiation refer to the so-called “gateway effect.” The included papers did not reliably establish a causal association between vaping and smoking initiation. Many papers referred to a so-called "gateway effect" as if supported by data, when it is not. Several such papers had an alarmist tone, lacked meaningful metrics, and lacked relevant descriptions of vaping-related behaviors. As such, the authors' conclusions were unreliable.

Some papers investigating health outcomes as a result of vaping attempted to identify effects of vaping in a cohort of former smokers, with significant pre-existing health conditions that would mask assessment of the outcome of interest.

Study designs should carefully consider the causal pathway under study. Without a longitudinal study design, it is difficult to infer causation, yet cohort studies suffer their own problems when studying vaping due to issues such as the stock-flow problem. Careful attention should be given to account for the numerous confounders. However, in real-life settings, where it is not feasible to conduct research under randomized, blinded, controlled settings, the prospect of residual confounding is very likely.

Overall, the population-based studies lacked granularity and meaningful metrics, and therefore, could not reliably make causal claims.

Some studies contained interesting data points worthy of future research, but lacked generalizability beyond conditions specific to the study. The lessons available from the papers in this review are predominantly negative. There are several papers that are solid workaday building blocks, but their generalizable lesson simple: “don’t overreach.” Most of the included papers offer only errors from which to learn. The questions most researchers address are far more difficult than typical epidemiology questions. We found no studies that employed carefully designed, fit for purpose methods to try to address the particular challenges of answering these difficult research questions. Our analysis provides several specific lessons:

First, none of the included papers proposes a valid hypothesis, and none assesses what associations we should expect to find if truly based on causal pathways. Determining causal association is very complex, particularly in the context of vaping and smoking behavioral research. This research study designs do not account for such complexity.

Second, changing the exposure and result measurements from "vaped at least once in the last 30 days and smoked every day for the last month" to, for example, "vapes daily and smoked at least once in the past week" would be more relevant for public and clinical health purposes.

Third, proposed causal claims must be made precisely, in terms of exposure(s) and outcome(s), and with hypotheses about the various potential causal pathways. The research should then be designed to assess whether the results support the primary hypothesis of interest. Attention to causal pathways would avoid many of the problems noted in the included papers. However, we acknowledge the challenge of addressing multiple causal pathways that would produce a particular association, and the difficulty in distinguishing them, as is the case with the “gateway” studies.

Fourth, it is important to recognize the pathway that vaping inspires additional quit attempts that would not otherwise happen. Overlooking this pathway is a common failure in research design. The stock-flow problem could be avoided by recognizing, for example, that the pathways to smoking plus vaping at a particular point in time include discovering that vaping is not a satisfying complete substitute, while the pathways to being among non-smokers includes discovering that it is.

Fifth, causal pathways are most often considered in research with regard to confounding and identifying which variables should and should not be used as control covariates. The use of causal pathway analysis would also be useful in these areas of research, but was not contemplated in the papers we reviewed.

Finally, using conventional epidemiology methods to assess complicated causal questions is not appropriate for real-world science such as vaping. For example, trying to identify health effects of vaping in a cohort of former smokers is quite challenging, as it is nearly impossible to reliably distinguish what can only be a tiny signal from enormous noise. This is made worse by flawed measures of smoking history and vaping patterns.

One of our aims in preparing this analytical review was to identify common, avoidable methodologic mistakes and to provide simple lessons for conducting more robust research. Perhaps, an important lesson is to identify important relevant research questions. Useful questions are those that are precise, contingent, nuanced, and focused on quantifications that are motivated by externally defined questions rather than what is convenient to do with a dataset.

Another aim was to empower readers of vaping literature to critically analyze the studies, findings, and conclusions of papers they may read. Skepticism as to the validity of conclusions may be warranted, because they are often misleading and unsupported. This is not currently a field where high levels of trust in “the scientific literature” is warranted and where readers would be able to extract reliable information without consideration of the methodological issues pointed out in this review.

Readers, however, should be able to come away from reading the present paper with a better collection of ideas about how to assess what research results really show, and whether the authors' claims are accurate. The findings from the studies included in our review, alongside more general reading, has been formulated in Table 2 into a series of recommendations for future research in the field of vaping and smoking cessation, initiation and health outcomes.

It is also worth noting that due to the slow pace of both data releases and the publishing process, alongside the rapid change in vaping technology, almost all publications in the journal literature are out of date by the time they are published. In an area of study where technology is improving year-to-year, fads and social acceptability have changed multiple times, and dominant messaging can change month-to-month, timing is important. Non-academic literature and pre-prints may then be a useful adjunct to the more formal peer-reviewed journal articles and should be included in the arsenal of evidence base.

The findings of our analysis have implications for researchers, reviewers, and scientific editors; we have distilled out clear recommendations for optimal, if aspirational, study design and analysis strategies for assessing the impact of vaping on the outcomes of interest into a summary table (Table 3). It is the authors’ view that this would further strengthen the paper by providing a useful and readily accessible template for investigators to refer to in planning their own vaping-related studies as well as for readers and policy makers to help them in evaluating the validity of the literature on vaping.

**Table 3:** Summary table of key study design recommendations

Area of study	Recommendation	Comment
Impact of vaping on smoking cessation/reduction	Ensure allocation and randomization of research participants to make sure to avoid selection bias, including (if possible) analyzing of those who have already quit by vaping	Limiting the enrollment to a subgroup of smokers (e.g., active smokers) omits those who had successfully quit by vaping, and prevents gathering generalizable findings
	Consider possible causal pathways towards smoking quitting/reduction, which could be attributable to vaping initiation	Data collection and analysis should be designed to investigate the quit/reduction attempts attributable to vaping, particularly in individuals with a history of previous unsuccessful attempts and “accidental quitters.”
Impact of vaping on smoking initiation	Detail vaping (and smoking) habits and history, in terms of their duration, amount, and frequency	The phrases “tried vaping” or “was a vaper” are limited as proxy indicator of levels of vaping exposure, and unreliable to support the gateway claim
	Analyze population-level trends in vaping incidence and prevalence together with smoking trends, and “triangulate” the findings across multiple types of evidence	“Gateway” studies are inconsistent with actual population-level trends, as “gateway” hypothesis would predict more smokers, but population-level trends show *faster* declines in smoking. Thus, the role of vaping in *preventing* smoking initiation should be considered
Health outcomes	Acknowledge the health consequences of previous smoking history in the evaluation non-acute effects of vaping, accounting for duration of smoking, time since quitting, or frequency and quantity of tobacco use	The vast majority of vapers are former smokers, and possible health events should be weighted as a function of previous smoking exposure (in particular for those conditions whose onset continues for longer after quitting)
	Ensure temporal relationships are consistent with the association being tested	Outcomes cannot logically occur *before* the exposure being tested, yet such data points are often included, especially in cross-sectional studies
General	Specify the exact causal pathway being tested—including particular exposure, outcome, and potential mediator variables—and think through plausible causal mechanisms	Different causal mechanisms are involved for outcomes of experimentation vs. regular use. Also, an association could include possible multiple mechanisms, specify which one is being tested (e.g., vaping impacts on *number* of quit attempts vs the *success* of each quit attempt). For health outcomes, specifying the exact causal pathway can inform about biological plausibility
	Use sufficiently robust methods to measure and control for relevant confounding factors, including multivariate statistics and analyses, and propensity score techniques if possible	All studies should control for detailed smoking and/or vaping history (frequency, quantity, duration of use). Additionally, cessation/reduction studies should control for number and methods of quit attempts, goals, etc.; initiation studies should control for peer and family tobacco use, personality characteristics, mental health factors, demographic characteristics; and health outcome studies should control for other relevant environmental or exposure factors
	Ensure that implications and conclusions do not assume a *causal* relationship, unless causality has been established	Implications that suggest altering one variable to change another, assume a causal relationship; this is inappropriate when only an association has been established
	Discuss biases, limitations, and alternate explanations honestly and transparently, and discuss how they impact findings	Confounding is a serious limitation in observational studies, and can render the entire set of results inconclusive. Biases from sample definition should be discussed (e.g., omitting those who previously quit by vaping). Alternate explanations such as diagnostic bias and reverse causality should be discussed

The findings of our review article have implications for policy makers. Our review found that very few studies were sufficiently rigorous to form conclusions on smoking cessation, initiation (the so-called Gateway Effect) and health risks and were not rigorous enough to inform policy. Yet such studies are used for policies (e.g., on tobacco harm reduction regulation). Policies should be informed by better science; this review and two previous systematic reviews on this topic support a more rigorous approach by policy makers in the selection of studies used to inform policy [2, 32, 33].

## Conclusion

Our critical appraisal reveals common, preventable flaws, the identification of which may provide guidance to researchers, reviewers, scientific editor, journalists, and policy makers. One striking result of the review is that a large portion of the high-ranking papers came out of US-dominated research institutions whose funders are unsupportive of a tobacco harm reduction agenda.

However, this does not mean there is a trove of good research out there that answers the big questions, but merely did not make the popularity cut. There is not. Notably, papers discussing the effect of vaping on smoking initiation shared common flaws. By contrast, papers addressing the effect of vaping on smoking cessation or reduction demonstrated a broader variety of flaws, yet common themes emerged. Our analysis of common flaws and limitations may guide future researchers to conduct more robust studies and, concomitantly, produce more reliable literature. There are countless sources of good building-block information that can be pieced together to provide knowledge. To provide useful information, research questions should be precise, contingent, nuanced and focused on quantifications that are motivated by externally defined questions. Such research necessitates proactive design, rather than utilizing already existing, but not fit-for-purpose, datasets.

## Appendix A

### Impact of vaping on smoking cessation and reduction

The specific strengths and limitations of studies examine the effect of vaping on smoking cessation and reduction are the following:

#### Gomajee et al. (2019) [15]

The authors examined a large nationally representative cohort in France with recruitment starting in 2012. The cohort included 5400 smokers and 2025 former smokers with an average follow-up of two years. Data collection was not limited to recent e-cigarette use, but took also into account when someone started vaping regularly.

The results suggest that regular e-cigarette use significantly reduces the quantity of cigarettes consumed and increases cessation. A significant increase in the rate of smoking relapse is observed among former smokers.

Although there are no apparent major flaws in this study, a number of identifiable imperfections are noted: (1) insufficient propensity covariates (unable to control for various generic causes of confounding); (2) no sign they actually thought at all about causal pathways, regarding confounding or the causal hypotheses; (3) authors fail to attempt to measure how many people started vaping and rapidly quit smoking entirely—they have the data to be able to do this, but they just throw away the opportunity to measure it.

For the present analysis what is relevant is that these findings are plausible rather than being obvious artifacts of methods problems. If the literature on the topic looked like this, we would be well informed.

#### Hitchman et al. (2015) [17]

This was a two-stage cohort study in Great Britain, starting in 2012 (a time when vaping was already fairly well established there), that recruited people who smoked at baseline, asking if they vape and measured vaping and smoking status a year later.

Although using appropriate methods for the analyses, this study has a stock-flow problem, a significant issue for most of the prospective cohort studies investigating the effect of vaping on smoking cessation and/or reduction. It selectively excludes most of the people who successfully quit smoking by switching. Any cross-sectional collection of people who currently smoke (in a population where vaping is already established) will exclude anyone intending to try to switch to a vaping product or intentionally trying to stop smoking by using e-cigarettes and succeeding. It will also exclude anyone who tried vaping without the intention to switch but liked it so much they switched anyway. The stock-flow bias would be reduced (though far from eliminated) by looking at just those subjects who tried vaping first during the follow-up period. The reported results show many more vapers at follow-up than at baseline. Looking at whether just these people were more likely to have quit smoking than those who had not tried vaping ever would have come much closer to addressing the main counterfactual of interest than anything that was reported.

This type of problem makes a cohort study an inherently an inappropriate study design for answering the main question of interest, unless it starts while vaping is still rare in a population or captures retrospective data and includes people who already quit smoking (effectively incorporating elements of a good case–control study, a study design that avoids this particular problem).

In the study, people who used closed-system e-cigarettes were less likely to abstain from smoking than non-vapers. Those who vaped open systems daily were considerably more likely to have become abstinent. People who used open systems less-than-daily were less likely. When discussing the implications of these associations, the authors suggest that the only possible causal story is that the vaping behaviors caused the different smoking outcomes.

It is possible that if someone invested in an open system and used it daily, then that might have caused them to quit smoking. But it is definitely the case that someone who quit smoking is more likely to have vaped daily (rather than less than daily). People who want to consume nicotine every day but have not quit smoking do not need to vape every day since they still smoke. It is possible that if someone invested in a good quality open system and used it daily, then that might have caused them to quit smoking. People who want to consume nicotine every day but have not quit smoking do not need to vape every day since they still smoke, or they might consider to complement with a cheaper and compact closed system to be used anytime anywhere.

The authors include in their model a collection of covariates related to propensity to quit smoking. While it seems reasonable to include all these variables, it is a mistake to include them in the analysis without giving serious consideration to their potential causal role. However, this along with technical problems like the non-representative recruitment method and high loss-to-follow-up are relatively minor compared to problems related to stock and flow and reverse causation.

#### Biener and Hargraves (2014) [8]

This study was based on a 2014 follow-up of a representative sample of adults in two US cities who smoked in 2011/2012. The authors emphasized the result that those who reported intensive (daily) vaping at follow-up were much more likely to have quit than those who did not vape at all, while those who vaped intermittently were much less likely to have quit. The careful assessment of frequency in e-cigarette usage in this survey is a good starting model for other researchers designing surveys.

The authors conclude that the associations are causal in the direction of the vaping behavior causing the smoking cessation outcome. Nonetheless, alternative causal pathways should have been considered. For example, results could have been explained by the fact that quitting smoking causes someone to be more likely to vape every day rather than less often (if they quit by switching). Furthermore, quitting smoking causes some people to not vape at all (if they chose to become completely abstinent), whereas they might have vaped occasionally as part of their smoking routine had they not quit.

In the study most people who smoked were aware of e-cigarettes and many had tried them, and thus we can conclude that many of those who were inclined to switch to vaping would have already done so, thus creating the stock-flow bias.

Last but not least, it is possible that the low retention rate (51%) at the follow-up interview could have introduced modifications in the study sample with significant impact on reported results.

#### Grana et al. (2014) [16]

This brief, one page article describes a one-year follow-up of a US cohort study, which began in 2011. The data and analysis appear flawed, and the study design did not account for the stock-flow problem, thereby introducing serious limitations. Second, participant traits in the sample population suggest that the researchers did not appropriately consider inclusion/exclusion criteria, and instead introduced bias into the study. Third, the protocol adopted an inaccurate data collection method (it is unclear on what criteria authors discarded 20% of the data). These limitations were not accounted for in the data interpretation or discussion, and the conclusions appeared to be unreliable and misleading.

#### Martínez et al. (2020) [20]

The authors followed a population of dual users “who were not necessarily seeking smoking cessation treatment” (2016–2017, across the USA, mainly online recruitment). Their primary analysis is about smoking reduction among vapers, and they acknowledged that they needed to collect retrospective data to assess that. The paper shows that dual use leads to a marked reduction in the number of cigarettes consumed per day.

This was a secondary analysis of current dual users, which did not include vapers who had already quit smoking. Therefore the authors were unable to compare dual users versus successful quitters and noted this as a limitation in their paper.

There are two stock-flow issues with this paper: "accumulated stock" and "rapid transit". The accumulated stock problem occurs when your baseline population includes years’ worth of people who tried vaping and did not find it helped them quit smoking, and thus it probably will not ever. The rapid transit problem is that most people who are successful exit the at-risk population quickly and so any purely cross-sectional collection (as at baseline in this quasi-cohort study) will miss most of them. These obviously affect the calculation as most of those who were going to reduce smoking as a result of vaping already did so.

Although reporting that the onset of vaping was associated with increases in self-reported nicotine use and dependence, authors are pretty clear at not inferring causality from the reported associations.

The lack of standardized metrics to define nicotine use and dependence in e-cigarette users represents a serious issue with the nicotine literature. It turns out that these comparisons were driven mainly by the number of “vaping sessions” per day compared to previous smoking sessions. As is often the case, metrics that are valid and reliable for the assessment of combustible cigarette nicotine dependence may not be valid and reliable for the assessment of electronic cigarettes. Whilst smoking sessions almost always consist of one whole cigarette, for obvious reasons, a vaping “session” is characterized by different patterns. While increases might have occurred, the measures used in this paper are not designed to show that. The authors acknowledged that this is an important limitation.

#### Gmel et al. (2016) [13]

This study followed 5128 20-year-old Swiss men, with a baseline survey (sometime between 2010 and 2012, at their induction into mandatory military service) with an average follow-up of about 1.3 years. At that time, vaping was relatively rare in Switzerland, and sales of nicotine-containing e-liquid were banned. The vaping exposure measure was any consumption in the last 12 months at follow-up only. Smoking exposure also was last 12 months, but was done at baseline also and included a frequency measure.

This study shows that vapers were more likely to be smokers (as shown in many other studies). Vapers reported more additional quitting attempts than non-vapers, but were less likely to quit smoking.

Unfortunately, the study design leaves us incapable of learning much more than that. The successful smoking cessation (between baseline and follow-up) results are difficult to interpret due to a combination of factors: the majority of vapers were probably vaping non-nicotine e-liquid, the fact that occasional vaping is frequent among regular smokers, and the stock-flow problem (because only the people who smoked were asked about vaping, so anyone who already quit smoking with vaping were not included among the vapers). Authors could have avoided the stock-flow bias by simply asking former smokers (at baseline) if they vaped previously to get an idea of how many switchers had already switched before baseline.

That vapers were more likely to be smokers that smoked more cigarettes is justified by the notion that people who are more dedicated smokers have a propensity to try nicotine/vaping products and therefore, it is not surprising to find that they are going to be more likely to vape. This type of analyses do not teach us anything new.

#### Etter and Bullen (2014) [11]

This paper used a rolling worldwide convenience sample of people who vaped (volunteers recruited via internet retailers and vaper social media), collecting baseline information from 2010 to 2013, with follow-up surveys one month and one year after that. Unsurprisingly, given a sampling method which almost certainly selected for more dedicated vapers, a large portion of subjects were still vaping at follow-up, and a large portion of those who still smoked at baseline had quit. However, since the sampling properties are unknown, there is no way to generalize this beyond the survey population. The authors emphasize a claim that vaping prevents “relapse” to smoking. But because the properties of the sample are unknown, it is impossible to estimate what the baseline rate of resuming smoking would have been, without vaping and to reasonably make that claim.

The key lesson here is that it is possible to derive reasonable information from almost any data. The questions being addressed need to be tailored to the limitations of the data. Authors could have investigated whether a particular variation on vaping behavior was associated with an outcome within the sample. Beware, results can only be generalized to the type of people who volunteer for a survey like this.

#### Warner (2016) [26]

The author looked at the 2014 Monitoring the Future (MTF) survey, a representative survey of 12th graders. The reported results focus on how smoking status was associated with vaping. The results and discussion are about how almost all vaping was concentrated among smokers and was rare among never smokers. Considering the cross-sectional nature of the study and the lack of any information about temporality of smoking and e-cigarette use initiation, the author is careful not to leap to the conclusion that this shows that most teenage vaping is a promising method for teenage smokers to quit or reduce smoking, but notes that this is possibly true.

The analysis provides useful insights, addressing the issues of different measures of usage, and how they have different implications and results. A major strength of the study is that authors did not define e-cigarette use in dichotomous terms (past 30 days vs. no use) but considered the frequency of use for both e-cigarettes and tobacco cigarettes. It has been shown that the definition of current e-cigarette use (i.e., any past 30-day use) includes a lot of experimenters who are infrequently using e-cigarettes. This too contrasts with many similar papers, in which a single “any use” measure of vaping status is used without any acknowledgment that other measures could be used and that a particular measure may not work for the causal claim being presented.

Another important finding of the study was that e-cigarette use was mostly confined among adolescents with a smoking history, while use by never smokers was rare. However, smoking frequency and intensity did not appear to correlate with vaping. Figuring out if this is a robust and generalizable relationship and exploring why it happens could be useful.

#### Giovenco and Delnevo (2018) [12]

This large (*n* = 15,532) cross-sectional study looked at people who currently smoked or had recently quit smoking (4–5 years before participating to the surveys). They examined a population-representative sample of the US population by merging the 2014 and 2015 National Health Interview Survey (NHIS). The time-point for former smokers was 2010, since that was the time that e-cigarettes became popular and widely available. The authors reported that vaping daily was strongly associated with being recent former smoker rather than current smoker. Occasional (less than daily) e-cigarette use was associated with being a current smoker. The authors made no claims about causation, as expected when analyzing cross-sectional surveys.

Another conceptual and methodological superiority of this study over most others is that it does not merely pick one measure of the vaping exposure, but contrasts the results for use frequency. It is expected that infrequent e-cigarette use will unlikely satisfy smoking craving or serve as a complete smoking substitute. Additionally, using e-cigarettes as an aid to quit smoking would imply regular use, a pattern similar to smoking. As the authors note, there may be a lot of occasional vapers who are just vaping to deal with smoke-free situations. Experimentation or use out of curiosity may be other reason for e-cigarette use that might conflict with the assessment of their effect on smoking cessation. Another possibility for current smokers who occasionally vape is that they may have tried to switch but realized e-cigarettes were not satisfactory enough and ended up using them infrequently.

Another major point in this study was that included recent former smokers. This is justified considering that the population of former smokers is heterogeneous and may include people who have quit long before e-cigarettes became available or popular. Including these people in a study assessing e-cigarette use means that the results would be skewed towards not showing an association because of the bias related to including former smokers who could not have quit with e-cigarettes due to unavailability. This may dilute or even mask any potential association between e-cigarette use and being a former smoker.

The study also tell us that examining all ever-vapers as a homogeneous group is inappropriate. Ever vapers is a largely heterogeneous population, lumping together fundamentally different (and measurable) behaviors and motivations for use, and then calculating a meaningless weighted average of how the different behaviors are associated with an outcome.

While the authors acknowledge the limitations of the cross-sectional design by addressing the possibility that the association between daily vaping and being a former smoker may indicate that people who had already quit smoking before e-cigarette use initiation subsequently became vapers, they pointed out the very small proportion of quitters of previous years (when e-cigarettes were not widely available) were e-cigarettes users. Thus, it is unlikely that e-cigarettes are attractive to already established former smokers.

#### Brown et al. (2014) [10]

This research was primarily descriptive epidemiology (a representative sample of smokers from Great Britain, 2012). The authors refrain from drawing causal conclusions, but the statistics collected better inform the causal questions of interest than many papers that purport to study causation.

The study looked at people who currently smoke or had quit recently (within the previous year). Their observations—when combined with an understanding of human behavior—are informative about potential causation or as building blocks. For example, among people who never vaped, those who had already quit smoking were far less likely to be interested in trying vaping. This is evidence of a causal pathway that is often ignored: people who have already quit smoking without using e-cigarettes are not particularly interested in a substitute. There was extensive awareness of vaping in 2012 and fairly good understanding it is lower risk compared to smoking. This implies that it is hard to assemble old enough data that the stock-flow problem can be overcome without retrospective questions.

## Appendix B

### Impact of vaping on smoking initiation

The specific strengths and limitations of studies examine the effect of vaping on smoking initiation are the following:

#### Barrington-Trimis et al. (2018) [5]

The authors used data pooled from three prospective cohort studies in California and Connecticut (baseline: 2013–2014; follow-up: 2014–2016) for older American teenagers (*N* = 6258) seeking for associations of e-cigarette use with smoking behaviors at follow-up.

Authors report empirical evidence by saying they are merely associations. Despite no attempt to assess whether the observed association is causal the conclusory statements are all based on assuming it is causal. The authors point out that some vapers did not previously smoke yet lead on to state that these individuals are most likely to take up smoking in the future. They also suggest that if vaping were curtailed there would be less smoking.

As for most papers in gateway literature, this also fails to consider the obvious confounding. The covariates used in the analysis (gender, race, and grade in school) do not control for obvious confounding. The associations between vaping and smoking are, thus, inevitable.

The dataset consisted of combining together measures from three different cohort studies, from three different places with hugely different demographics, from three different age groups and follow-up patterns, spread out over different time periods and using different measures of the “same” variables. The behavioral patterns observed in this particular study population are not universal constants, and findings do not apply to all periods and populations.

The authors do not report their statistical or categorization methods in enough detail, nor they explain how survey questions were asked and in what order. As a result of the methods being so unclear and the data being a miscellany of several distinct populations, it would be almost impossible to make sense of the results aside from the confounding problem. Perhaps, these data could have been used to compare differing associations across strata within the pooled data (e.g., those who had only tried vaping versus dedicated vapers—but data were combined).

#### Leventhal et al. (2016) [18]

This research letter used data from a 6-month follow-up of 10th graders in Los Angeles (USA). Authors concluded that teens who used e-cigarettes became “heavy” smokers by showing an association with higher level of vaping intensity (with the top category, “frequent”, being merely vaping three days in the past month) and the outcome of “frequent” (three times per month) and “heavy” (two cigarettes per day on smoking days) smoking. Atypical definitions for the exposure and outcome variables were used. Also, whilst past and never vaping were separated, past and never smoking were combined. These decisions should be justified as they impact on the results.

The main problem (common to most gateway literature) is the uncontrolled confounding and the failure to examine it. The analysis includes de-confounding covariates that could conceivably adjust away half of the association caused by confounding, but there is no possible way for them to eliminate all of it. There is a common misconception that a dose–response relationship is suggestive of causation rather than confounding, but confounding often has a dose–response relationship too. Consequently, the reported association can be also interpreted as having a greater taste for nicotine will cause the propensity to vape more and to smoke more, but that tells us nothing about causation. It is expected to find associations between vaping and smoking when relevant confounders are not factored in.

#### Bold et al. (2018) [9]

This prospective study enrolled high school students from 3 public schools in Connecticut (USA) and followed them up cross three longitudinal waves (2013, 2014, and 2015).

The authors looked at various patterns of consumption of cigarettes and e-cigarettes and found an association between vaping and smoking behaviors at follow-up.

Although a good number of covariates were included in the analysis, it is unlikely that the study was adequately controlled for different propensities. Therefore, there is no way causal conclusions can be made based on this data.

The authors, in reporting an upward trend across survey waves in e-cigarette consumption prevalence and quantity, attribute this to an alarming social secular time trend, despite also acknowledging that this is likely to result from their study subjects getting older.

The choice of exposure and outcome measures illustrates an additional flaw; all measures are dichotomous measures of having used the product, even just once, within the past month at the time of each of the three survey waves (they had other measures but chose this one). Using such a measure means that many of the “gateway” events might be occurring among subjects who already smoked. Someone who vapes but does not smoke during a given month will already be a casual smoker who simply did not smoke that month (perhaps because they had a supply of vapes and no supply of cigarettes at the time). Worse still, those who smoked in the previous period seem to be included in the “vaped and then later smoked” outcome.

As is inevitable for this type of study design, vaping in one period was associated with smoking in the next. The authors slip into assuming this means vaping is causing smoking without the intermediate step of even saying they are concluding that, calling for anti-vaping measures to reduce smoking and suggesting that the only unsettled questions are why their assumed causation happens.

The authors look at something other than a single association to try to support their causal claim, but what they do is incorrect. They suggest that because they claim smoking at one wave did not predict vaping at the next wave, that this somehow supports their gateway conclusion. But the claim is false—they also found a strong association in that direction, as is inevitable. To authors credit, they are clear that their results “may” not generalize beyond a high SES white population in one place in the USA.

#### Goldenson (2017) [14]

This prospective study enrolled a small group of students from 10 high schools in the Los Angeles (CA) metropolitan area and followed them up 6 months later. The study found that California teenagers’ choice of nicotine strength in their vapes is associated with subsequent smoking at follow-up.

The results are driven by a questionable parameterization of their data (forcing their model to assume that each step from one of their arbitrary nicotine strength categories to the next will always have the same effect on the outcome). There are barely noticeable changes in the univariate statistics and crosstabs, from baseline to follow-up. Yet these became dramatic ratios in their multivariate model, and this disconnect is not acknowledged or explained by the authors.

De-confounder variables in this analysis include some “risk taking” index variables and one rough SES measure, but there is no reason to believe this could control for propensity.

The usual rhetoric of addressing associations and then slipping into assuming they are causal is present here. Given the significant associations found in this study, the possibility of alternative explanations should have been considered.

The main problem in this paper is that most of the subjects who reported vaping higher nicotine concentrations were already smokers at baseline. It is their greater prevalence and intensity of smoking at follow-up that drives all the main results. There was an increase in the prevalence and intensity of smoking, from baseline to follow-up, for the highest-nicotine vaper group, but it was modest. In effect, they started with an exposed group that already had the outcome and then suggested that the exposure resulted in the outcome at follow-up.

The only apparent useful take home message is that some people like to consume nicotine a lot, some do not like it and others are in between. The same group who vaped high nicotine also smoked, and smoked more. Meanwhile, those who vaped zero-nicotine or low-nicotine were unlikely to smoke or to start smoking, and smoked a bit less at follow-up, rather than more. Authors interpret this as vaping higher nicotine causes smoking, but dismiss other more plausible explanations; for example, that people who like consuming nicotine a lot like products that deliver a solid dose of nicotine more than do people who do not like nicotine.

#### Unger et al. (2016) [25]

This cohort study followed up Hispanic teenagers into young adulthood, in Los Angeles.

Although the focus was on smoking and cannabis use, the study added questions about e-cigarette use in the last two waves in 2014 and 2015, when the participants were approximately 25 years old (*n* = 1332).

Failure to control for relevant confounding (the only covariates were a few simple demographic variables, use of alcohol, and use of other tobacco products) resulted in the expected associations between vaping, smoking, and marijuana use.

The authors discuss these associations as if it were unequivocally causal, without discussing any other possible pathways that could explain the relationship. Moreover, the only usage data were the dichotomous “any use in the last month”. Someone who experiments with tobacco/nicotine product use, vaping sometimes and smoking sometimes, would be a “gateway” case if they happened to have vaped but not smoked for one month in 2014 and happened to have smoked during one month in 2015. Everyone with no inclination to use tobacco products would, of course, contribute to the denominator of non-vapers who did not “start smoking”. That makes this an exacerbation of the main confounding problem (that some people like nicotine while others do not), made worse by the choice of exposure definitions.

This study also reported an analysis showing that vaping was not associated with smoking cessation between the two waves, but result were based on a very small effective sample size.

#### Gmel et al. (2016) [13]

This study, described in the previous section, followed 5128 20-year-old Swiss men with an average follow-up of about 1.3 years and found that vapers were more likely to be smokers.

The measure of smoking initiation in the study was “had not smoked (at all) in the year before age-20 baseline, but had in the year before age-21 or -22 follow-up” with the exposure of interest being “vaped (at all) in the last year before follow-up”. The result was the inevitable strong association due to the obvious confounding problems, which are not acknowledged or substantially controlled for.

Measuring vaping exposure only at follow-up exacerbates the difficulty in interpreting the smoking initiation results. Did someone who smoked for the first time during follow-up try both smoking and vaping for the first time, or were they already vaping and (according to the implicit story) were caused to smoke as a result? We do not even know in what order the two items were first evaluated.

#### Spindle et al. 2017 [24]

This study is two-period cohort with 3.757 students from a mid-tier college in Virginia USA, surveyed in 2014 and again in 2015. The study found an association between never-smoking participants who had tried e-cigarettes at baseline and cigarette use a year later.

This survey seems to have better deconfounder variables for “risk taking” inclinations compared to most contributions to this scientific literature, but nothing to control for having a taste for nicotine or an aversion to nicotine products. The consequential probability of the fatal confounding problem results in the inevitable association. The authors just assumed that all associations represented causation in a direction they preferred to believe, without considering the analysis of possible reverse causation pathways.

The emphasized results are for subjects who reported that their first ever use of a product was during the follow-up period, avoiding the alternating experimenting problem. It seems odd, however, that 30% of the ever-vapers at baseline made the rather challenging transition back to never having vaped in their lives at follow-up; a result that appears in a table and suggests some data quality problem probably due to recanting of e-cig/cigarette use. Recanting is commonplace in longitudinal studies, particularly those with adolescent and young adult samples, and may occur due to a variety of reasons (e.g., social desirability, recall bias) (see ref Fendrich, & Rosenbaum, 2003). That one of their key input variables was demonstrated to be wrong 30% of the time is problematic. Difficult to draw key conclusion when data are unreliable. The authors acknowledged this as a limitation in the paper.

#### Miech et al. (2017) [22]

This paper is based on a relatively small sample (*n* = 347) from a US national survey of 12th graders surveyed in 2014 and resurveyed again one year later. The study found an association between never-smoking youth who had tried e-cigarettes at baseline and past-year cigarette smoking a year late. An association was also found between occasional-smokers who had tried e-cigarettes at baseline and past-year cigarette smoking at follow-up.

Compared to similar papers, it shows more scientific sophistication and avoids some of the fatal errors. By looking at never smokers at baseline, authors have eliminated some of the fatal flaws of similar studies in this category. They also reduced the susceptibility variations in the population by restricting one of their analyses to subjects who reported a belief that smoking poses “great risk”. Yet, they had obvious uncontrolled confounding (they had only a handful of demographic covariates), and thus, the association remained inevitable.

The authors make clear they understand the concept of causal pathways, but they eventually fail to really discuss the implications and make the mistake of assuming the usual inevitable association represents vaping causing smoking without examining other reverse causation pathways that could explain the association. Causation in the “wrong” direction, plus some of the inevitable random drift in responses to a vague “feelings” question, could explain the entire reported result.

#### Primack et al. (2015) [23]

This study is a two-period follow-up cohort study, of 694 young (16–26 years) US never smokers, with baseline survey during 2012–2014 and follow-up a year later. Included subjects were “non-susceptible” never smokers, defined as asserting “definitely no” when asked each of “If one of your friends offered you a cigarette, would you try it?” and “Do you think you will smoke a cigarette sometime in the next year?” While it would be important to define non-smokers who are non-susceptible to initiate smoking in the future, it is highly unlikely that the approach based on two simple questions is enough to correctly capture “susceptibility” of this population subgroup.

While the study portrays vaping as a gateway to smoking even to people who are not susceptible to initiate smoking, there are several additional limitations. A very small proportion of participants (2.3%, *n* = 16) reported e-cigarette use at baseline, and this group was compared with the rest of the participants (*n* = 678). Thus, the study suffers from the problem of a small effective sample size for its main outcome. The small sample size resulted in large confidence intervals in the analysis.

The gateway theory can be challenged using the common liability model. According to this model, it is the overall susceptibility and tendency of individuals to engage into risky behaviors that dictates the use of multiple products. This model can explain several correlations between use of different substances (e.g., cigarettes and other tobacco products, alcohol, marijuana and drugs), and the bidirectional association between smoking and e-cigarette use. Finally, the gateway theory cannot explain the sharp drop in smoking prevalence among US adolescents from 2011 to 2020, a period where e-cigarette use (mostly experimentation) has grown considerably. Thus, along with the possibility that e-cigarette use may “cause” smoking among adolescents, an alternative possibility is that e-cigarettes may lead smokers away from tobacco cigarettes or may prevent smoking in adolescents who would have smoked had e-cigarettes not been available. These alternative possibilities were largely ignored in the discussion.

Notably, the study did not explain how e-cigarette use at baseline was defined. Thus it is unclear if the authors referred to ever, current or any other frequency of e-cigarette use. It is equally unclear how tobacco cigarette use was defined. But the real problem is ignored confounding. The control covariates were better than many other similar studies, but not good enough.

#### Chatterjee et al. (2018) [6]

This is a search using PubMed, Google Scholar, Scopus, and Web of Science in February 2016 to include longitudinal studies with data on e-cigarette use and conventional cigarette smoking among adolescents and young adults. The search identified four studies of the gateway effect.

Authors claim this is an analytic review of literature which qualifies under our inclusion criteria, though they get very little credit for doing any actual analysis. The authors failed to note any of the fatal flaws common to the gateway literature, despite them being obvious to anyone who has any business doing an analytic review. Effectively, it just copy–pastes the abstracts from the original papers. None of the studies explored the possibility that the common liability theory can explain the behavioral transitions observed.

#### Levy et al. (2018) [19]

This paper examines the temporal relationship between vaping and youth smoking using multiple US data sets from the 2010s. Notably, the paper addresses the acceleration in the smoking decline during the period of growing e-cigarette use, which is unacknowledged in the “gateway” literature.

In the Introduction, the authors note the obvious inconsistency between the population statistics and the stated conclusions, They concentrate on a possible problem of temporality (smoking actually predating vaping) which is indeed a secondary flaw in some of the cohort studies, but not one of the main problems. As already mentioned, there is a bidirectional association between smoking and vaping.

A problem is that the data are for use prevalence, while the claims tend to be about uptake incidence. Hence, there are different hypotheses that call for somewhat different analyses. Fortunately for the populations and exposure in question (where incidence is inevitably recent), prevalence is a reasonably good proxy for incidence.

The biggest flaw in this paper is that it vaguely alludes to the flaws associated with gateway cohort studies, but then fails to tie them to the implications of the observations in the paper. For example, there is no mention of the common liability model as a logical means of explaining the complex interactions between different behaviors.

#### Beard et al. (2019) [6]

This is an example of an ecological study of vaping and smoking, looking at population prevalence and incidence statistics for England as a 2006–2017 time series. Ecological analyses are probably the most informative approach available. Because the authors have individual-level data but convert it to ecological data, they are able to estimate directly the population impact.

In this paper, one of the major confounding problems for individual associations, that most people who vape while smoking are particularly dedicated smokers, is transformed into a comparatively minor source of bias. For smoking cessation, ecological analysis also avoids the stock-flow problem by not restricting analysis to those who are vaping at a particular time, and the long time series available avoids the problem of missing those who were most interested in switching and so switched before the first data was collected. However, this still brings with it the complication of properly modeling the diminishing marginal “effectiveness” of vaping, as those who are the most promising candidates for switching are depleted from the at-risk population.

Because this is a solid analysis, it presents an opportunity to comment on the misplaced pedagogical priorities that exist throughout this literature (and many other related literatures). However, authors devote little attention to the methods questions of greatest importance, those having to do with causal modeling.

While the generalizability of the findings derived from population-level studies is undisputed, such studies usually fail to identify and focus on specific subpopulation groups who may obtain the biggest benefit from e-cigarettes as a smoking substitute. As a result, the impact of vaping may be diluted. In this study, however, this is addressed by focusing on the use of e-cigarettes during a quit attempt. Thus, it tries to examine use during the time-point of interest.

## Appendix C

### Impact of vaping on health outcomes

The specific strengths and limitations of studies examine the effect of vaping on health outcomes are the following:

#### Bhatta and Glantz (2019) [7]

This article was retracted, apparently due to issues surrounding the authors’ legal access to the data, not due to fatal flaws in methods and analysis [34].

Despite retraction, this paper continues to be rated as among the most popular (as measured by the Google Scholar algorithm); further, citations in both academic articles and political documents continue to occur. This paper examines the association between vaping and myocardial infarction, using a large population-representative longitudinal dataset. However, researchers fail to report that most myocardial infarctions occurred before the subjects started vaping. Researchers Rodu and Plurphanswat publicized the problem; successfully campaigned for the retraction; and conducted a new analysis categorizing myocardial infarction that occurred before vaping initiation as having occurred in non-vapers. Rodu and Plurphanswat found a strong protective association with vaping, a contrary to the prior researchers’ misleading claim.

#### Alzahrani, Pena, Temesgen, and Glantz (2018) [4]

The authors found that heart attacks were associated with vaping in a large representative dataset. This paper belongs to the misleading-by-design category as it looked retrospectively at heart attacks reporting that does not contain the data to check whether the heart attacks occurred before or after the subject started vaping. Simple demographics suggest that it is almost certainly true that occurred before for the majority of them. Using a retrospective dataset that lacked timing information cannot help establishing the effect of vaping on heart attacks. This erroneous approach simply leads to misleading results.

Probably, the biggest problem with this paper is the impossibility to assess effects of vaping in a population of former smokers (the vast majority of vapers are). All vapers who are old enough to have enough disease or mortality risk to provide useful data are former smokers, and it is impossible to sufficiently control for the residual health effects of the former smoking to be able to tease out the effect of vaping. The contamination of the dataset with former smoking is the most plausible reason for the association of heart attacks with vaping.

#### McConnell et al. (2016) [21]

This study used survey data of older high school students’ behavior and showed an association between self-reported past-year history of respiratory symptoms (coughing, wheezing) and e-cigarette use, which disappears when controlling for tobacco smoking and second-hand smoke exposure (well-known triggers of acute respiratory symptoms).

The statistical association per se cannot prove causation. If the candidate causal claim in the paper is that vaping trigger acute respiratory symptoms that would not have happened otherwise, the obvious study design would be based on individual exposure crossovers, preferably with serial clinical assessments. If the candidate causal claim is that vaping a substantial amount for a while causes chronic respiratory problems to develop, the study design would not be based on 18-year olds (whose historical exposure is necessarily minimal) and insignificant vaping exposure (whose frequency was reported to be as low as once or twice in the last 30 days). Such a trivial health exposure could not cause any biological outcome. If a research method suggests that a few puffs on an e-cigarette caused measurable health outcomes, then the problem is obviously with the method, not the exposure.

The reported associations for the exposures and the outcomes must consider other possibilities. For example, subjects with well-educated parents had more than double the rate of reporting respiratory problems, despite the fact that this characteristic almost certainly reduces harmful exposures and improves medical care and allergy management. Presumably, the “risk” is that these parents are more attentive to any particular level of symptoms, resulting in a diagnostic bias.

What is worse is that the reported associations for the outcomes and vaping (an exposure that could theoretically sometimes cause breathing problems, but which overwhelming evidence tells us must be rare) are about the same as those for smoking (an exposure that is known to cause a lot of breathing problems, both acutely and due to cumulative damage).

Dataset was treated as a single cross-sectional study despite it being the 12th-year wave of a cohort study, from 2014, thus failing to make most of the exposure and outcome data. The vaping exposure information may have only been collected in that last wave (this is not clear), but other information is historical. Specifically, some of the subjects undoubtedly already had their self-reported respiratory symptoms (coughing, wheezing) well before they started vaping.

There is another general problem with public health datasets of this type. For any particular outcome it is likely that input variables that should have been controlled for are missing. In this case, when assessing respiratory outcomes it is important to collect information that are likely to have an impact, such as allergy diagnoses, the circumstances in which the symptoms occur (e.g., seasonality, places, exercising) and occupational exposures. None was collected.

#### Wills et al. (2018) [27]

This cross-sectional random-dial phone survey examined respiratory health among e-cigarette users in Hawaii, USA aged 55 years and older. In multivariable analyses, no significant association was noted between e-cigarette use and self-reported chronic respiratory conditions (asthma as well as COPD) in the entire sample that included smokers (AOR 1.27, CI 0.96–1.67; *p* = 0.10); however, when the analysis was confined to non-smokers, a barely significant association was found (AOR 1.33, CI 1.00–1.77; *p* < 0.05). The study did not seem to control for relevant confounding (e.g., information that are likely to have an impact, such as family history of allergic diseases, passive smoking, or occupational exposures were not collected) or classify participants by relevant health status.

Besides the obvious limitations regarding inferences pertaining to causality from cross-sectional studies that provide no information on temporal relations, another major limitation of this study is the failure to obtain any information on “dose”, so that dose–response relationships could not be assessed. Notably, vaping behavior was defined as “one puff ever” (representing trivial exposure from e-cigarette experimentation) whereas smoking behavior in a population with a mean age of 55 years means that people have smoked cigarettes more frequently and for a longer duration (i.e., decades). Attributing respiratory damage from such a low level of exposure would suggest a strong negative acute and chronic impact of the vaping, which seems implausible. The association is obviously residual confounding from smoking. The paper could have reported how associations could change when different strata of former smokers (recency of quitting, intensity of use) are taken into account. It could have looked at the associations for actual vapers rather than only reporting results for all ever-triers, despite the small sample size. It could have assessed whether the subjects had symptoms before they started vaping.
