# Correction to: Analysis of common methodological flaws in the highest cited e-cigarette epidemiology research

**DOI:** 10.1007/s11739-022-03022-9

**Published:** 2022-06-15

**Authors:** Cother Hajat, Emma Stein, Arielle Selya, Riccardo Polosa, The CoEHAR study group, Salvatore Alaimo, Carmelina Daniela Anfuso, Ignazio Barbagallo, Francesco Basile, Sebastiano Battiato, Brahim Benhamou, Gaetano Bertino, Alberto Bianchi, Antonio G Biondi, Maria Luisa Brandi, Emma Cacciola, Rossella R Cacciola, Bruno Santi Cacopardo, Aldo E Calogero, Maria Teresa Cambria, Davide Campagna, Filippo Caraci, Agatino Cariola, Massimo Caruso, Pasquale Caponnetto, Adriana Ciancio, Fabio Cibella, Maurizio di Mauro, Jennifer di Piazza, Adriana di Stefano, Filippo Drago, Salvatore Failla, Rosario Faraci, Salvatore Ferlito, Margherita Ferrante, Alfredo Ferro, Giancarlo A Ferro, Francesco Frasca, Lucia Frittitta, Pio M Furneri, Antonio Gagliano, Giovanni Gallo, Fabio Galvano, Giuseppe Grasso, Francesca Guarino, Antonino Gulino, Emmanuele A Jannini, Sandro La Vignera, Giuseppe Lazzarino, Caterina Ledda, Rosalia Maria Leonardi, Giovanni Li Volti, Antonio Longo, Gabriella Lupo, Mario Malerba, Luigi Marletta, Guido Nicolosi, Francesco Nocera, Gea Oliveri Conti, Giuseppe Palazzo, Rosalba Parenti, Eugenio Pedullà, Alfredo Pulvirenti, Francesco Purrello, Francesco Rapisarda, Venerando Rapisarda, Renata Rizzo, Simone Ronsisvalle, Giuseppe Ronsisvalle, Martino Ruggieri, Maria Santagati, Cristina Satriano, Laura Sciacca, Maria Salvina Signorelli, Marco Tatullo, Daniele Tibullo, Venera Tomaselli, Vladislav Volarevic, Luca Zanoli, Agata Zappalà

**Affiliations:** 1 Public Health Institute, UAE University, Al Ain, United Arab Emirates; 2 New Haven, USA; 3 Pinney Associates, Inc, Pittsburgh, PA USA; 4 Behavioral Sciences Group, Sanford Research, Sioux Falls, SD USA; 5 Department of Pediatrics, University of South Dakota Sanford School of Medicine, Sioux Falls, SD USA; 6 Center of Excellence for the Acceleration of HArm Reduction (CoEHAR), University of Catania, Catania, Italy; 7 Department of Clinical and Experimental Medicine, University of Catania, Catania, Italy; 8 Institute of Internal Medicine, AOU “Policlinico-V. Emanuele”, Via S. Sofia, 78, Catania, Italy

## Correction to: Internal and Emergency Medicine (2022) 17:887–909 10.1007/s11739-022-02967-1

In the original publication of the article, the author name has been incorrectly published as Maria C. Santagati in the Review (2022). The correct name is Maria Santagati.

The original article has been corrected.

